# Psychometric Properties of the Gastrointestinal Symptom Severity Scale in a Sample of Adolescents and Young Adults

**DOI:** 10.3390/jcm13061662

**Published:** 2024-03-14

**Authors:** Agustín Ernesto Martínez-González, Néstor Montoro-Pérez, Agustín Wallace, Susana Pérez-Sánchez, José A. Piqueras, Lidia Infante-Cañete, Silvia Hidalgo-Berutich, Tíscar Rodríguez-Jiménez, Pedro Andreo-Martínez

**Affiliations:** 1 Department of Developmental Psychology and Didactics, University of Alicante, San Vicente del Raspeig, 03690 Alicante, Spain; agustin.emartinez@ua.es; 2 Department of Nursing, Faculty of Health Sciences, Person-Centred Care and Health Outcomes Innovation Group, University of Alicante, San Vicente del Raspeig, 03690 Alicante, Spain; 3 Department of Developmental and Educational Psychology, Faculty of Psychology, University of Malaga, 29071 Malaga, Spain; awallace@uma.es (A.W.); lidiainfante@uma.es (L.I.-C.); shidalgo@avanza-online.es (S.H.-B.); 4 Hospital Pediatric Service University General “Los Arcos”, Mar Menor, 30739 Murcia, Spain; susanaperezsanchez@gmail.com (S.P.-S.);; 5 Department of Health Psychology, Miguel Hernández University of Elche, 03312 Alicante, Spain; jpiqueras@umh.es (J.A.P.);; 6 Area of Personality, Faculty of Social and Human Sciences, University of Zaragoza, 50013 Teruel, Spain; 7 Department of Agricultural Chemistry, Faculty of Chemistry, University of Murcia, 30120 Murcia, Spain

**Keywords:** functional gastrointestinal disorders, gastrointestinal symptoms, constipation, pain, adolescents, young adults

## Abstract

**Background:** Functional gastrointestinal disorders (FGIDs) are a set of chronic or recurrent gastrointestinal symptoms (GS) with great psychobiological complexity. The appearance of FGIDs harms quality of life and drains medical resources. **Methods:** Psychometric properties of the Gastrointestinal Symptom Severity Scale (GSSS) based on Rome IV criteria were examined in a sample of 1247 individuals with typical development. Observations were randomly divided into two subsets, namely, subsample 1 (*n* = 624) and subsample 2 (*n* = 623). Exploratory factor analysis (EFA) was performed with data from subsample 1, whilst confirmatory factor analysis (CFA) was performed with data from subsample 2. Internal consistency of the scale was assessed for the whole dataset according to ordinal alpha, whilst four-week reliability was measured according to the intraclass correlation coefficient (ICC). Measurement invariance as a function of sex was also examined, and discriminant–convergent validity of the GSSS was examined through hypothesis testing. **Results:** EFA revealed a two-factor structure with a moderate percentage of explained variance (51.3%), whilst CFA exhibited an excellent fit of the data to the model. A one-factor CFA model demonstrated an acceptable but slightly lower fit. Internal consistency was moderate and test–retest reliability was deemed adequate. Metric invariance was demonstrated as a function of sex. Hypothesis testing demonstrated strong convergent–discriminant validity with measures of sensory sensitivity, obsessive–compulsive symptoms, and pain. **Conclusions:** The GSSS is a tool with acceptable and promising psychometric properties when administered to neurotypical adolescents and young adults. The self-report GSSS may promote better understanding of GS involvement in the gut microbiota–brain axis in the general population.

## 1. Introduction

Functional gastrointestinal disorders (FGIDs) are a set of chronic or recurrent gastrointestinal symptoms (GS) which are not explained by structural or biochemical abnormalities. Thus, a complex psychobiological interaction exists that is closely related to the gut-microbiota-brain axis in FGIDs [1,2]. Emergence of FGIDs is associated with lower quality of life and more frequent visits to the doctor [3]. As a consequence, they entail an increase in average healthcare costs in developed countries [4]. One third of clinical gastroenterology referrals in the general population are for irritable bowel syndrome (IBS), functional dyspepsia, and functional constipation [5]. Currently, FGIDs are detected and diagnosed according to Rome IV criteria (2016). These new criteria represent an improvement over previous criteria because they place a greater emphasis on the interaction between the brain and the gut. In this sense, functional digestive disorders have been redefined using new terminology, specifically, gut–brain interaction disorders [1,6].

Half of the general adult and adolescent populations may meet FGID criteria at any given time (about 40% of adults and between 9.9% and 29% of adolescents), with these being more prevalent in women [3,7,8]. Specifically, 0–45.1% of individuals suffer from irritable bowel syndrome, 0.2–6.2% suffer from cyclic vomiting, 31.3–86.9% suffer from functional constipation, 31.5% suffer from IBS with diarrhea, 1.4–12% suffer from IBS, 20% suffer from acid reflux, and 10–17% suffer from functional dyspepsia [7,8,9,10,11]. Furthermore, two-thirds of these individuals will experience chronic fluctuating symptoms [4].

Previous studies have indicated that there may be a relationship between sensory reactions (e.g., being picky about certain foods) and the appearance of GS and pain associated with the gut microbiota–brain axis [12,13,14]. Additionally, a series of mechanosensory circuits is present in the intestine and digestive tract that is involved in intestinal health [15]. Further, a relationship between sensory reactivity, pain, and GS has been found [12,13], and a relationship has also been found between obsessive–compulsive symptoms and GS [16,17].

Recent studies indicate that different prevalence estimates are produced as a function of whether Rome III or Rome IV criteria are used, making it necessary to elaborate instruments that address current criteria [6]. Validation studies with a sample of neurotypical adults have been conducted according to Rome III criteria, with samples comprising fewer than a thousand individuals (e.g., Gastrointestinal Symptoms Severity Index—GISSI [18]). On the other hand, some instruments have focused on measuring symptoms through information provided by caregivers (e.g., Questionnaire on Pediatric Gastrointestinal Symptoms–Rome III [QPGS-RIII]) [6], and other scales have been developed for a specific clinical population with FGIDs with very small samples (e.g., Gastrointestinal Symptom Rating Scale [GSRS] and Irritable Bowel Severity Scoring System [IBSSS]) [19,20]. A recent instrument, the Gastrointestinal Symptom Severity Scale (GSSS), has been developed according to Rome IV criteria for children, neurotypical adolescents, and individuals with autism [14,21,22]. Outcomes reported by previous studies indicate that this scale has adequate psychometric properties, good internal consistency, and adequate test–retest reliability [14,22]. The GSSS comprises a single factor [14,22]. However, some evidence indicates that it could be used as a two-factor instrument to assess abdominal pain and defecation disorders (e.g., abdominal pain, gas, constipation, etc.) and functional nausea and vomiting disorders (i.e., regurgitation, passage of ingested food, etc.) [14].

To the best of our knowledge, no studies currently exist that evaluate the psychometric properties of the GSSS in populations of neurotypical adolescents and young adults. Thus, the objectives of the present study are to: (1) investigate the structural validity of the GSSS using sequential analysis, including exploratory factor analysis (EFA) and confirmatory factor analysis (CFA); (2) assess internal consistency and test–retest reliability over a 4-week period; (3) examine the measurement invariance of the GSSS as a function of sex; (4) conduct hypothesis testing as a means of exploring discriminant–convergent validity of the GSSS; and (5) provide descriptive data following the administration of the GSSS in a sample of neurotypical adolescents and young adults.

## 2. Materials and Methods

### 2.1. Design

An instrumental study is presented that was conducted to validate the GSSS and test its psychometric properties [23] in a sample of Spanish neurotypical adolescents and young adults.

### 2.2. Participants

Participants were selected using non-probabilistic convenience sampling at four universities in Alicante, Elche, Teruel, Murcia, and Malaga (Spain). The selection process took place between October 2020 and February 2022. Eligibility criteria included: (1) aged 17 years and above, (2) adolescents and young adults with typical development, and (3) proficiency in the Spanish language.

### 2.3. Sample Size

According to Ferrando et al. [24] and Lloret-Segura et al. [25], a sample size of at least 500 cases is recommended for EFA (*n* = 250) and CFA (*n* = 250), even with well-defined factors and optimal conditions. The study sample included 1247 individuals.

### 2.4. Measures

- Clinical questionnaire of gastro-intestinal symptoms: This is an ad hoc questionnaire that was developed to examine gastro-intestinal disorders according to Rome criteria [1]. The tool consists of a series of questions regarding gastrointestinal disorders (e.g., diarrhea, abdominal pain, dyspepsia, gastroesophageal reflux, etc.) and family history.
- Gastrointestinal Symptom Severity Scale (GSSS): This instrument is based on Rome IV criteria [1] and consists of seven items pertaining to main gastro-intestinal symptoms (constipation, diarrhea, average stool consistency, stool odor, flatulence and gas, and abdominal pain). The instrument comprises an abdominal subscale (abdominal pain, gas, and constipation) and a vomiting and defecation subscale (vomiting, defecation in inappropriate places, diarrhea, and rumination). Items are rated along a four-point Likert scale ranging from 0 (none/nothing or this symptom does not occur) to 3 (very frequent and troublesome symptom). The GSSS presents adequate psychometric properties in individuals with autism and in neurotypical children and adolescents [14,22]. Internal consistency coefficients of 0.73 have been reported in children with typical development [22], whilst coefficients between 0.61 and 0.75 have been reported in individuals with autism [14].
  Two versions of the instrument are available, namely, a version for caregivers–professionals and a self-report version. The self-report version of the test was administered in the present study (identical to the version for children and adolescents up to 16 years).
- Pain and Sensitivity Reactivity Scale (PSRS): This scale evaluates reactivity to pain and sensory reactivity according to 50 items. It is composed of three dimensions: pain, sensory hypo-reactivity, and sensory hyper-reactivity. Hyposensitivity and hypersensitivity dimensions include tactile, olfactory, visual, gustatory, and auditory items. All items are rated along a four-point Likert scale ranging from 0 (behavior does not occur) to 3 (behavior occurs and is a severe problem). In addition, the PSRS includes a pain reactivity domain that comprises seven items. The PSRS is based on a theory elaborated by Miller et al. [26] that alludes to sensory modulation disorders that are characterized by three different patterns (hyper-response, hypo-response, and sensory seeking) in accordance with identified diagnostic nosology. Two versions of the PSRS are available, specifically, a version for caregivers–professionals and a self-report version. The self-report version was used in the present study. Cronbach’s alpha values were calculated to evaluate the internal consistency of the overall scale, and its subscales showed strong internal consistency in a neurotypical young adult population (PSRS-total = 0.92; pain = 0.79; broad sensory hypo-reactivity = 0.88; broad sensory hyper-reactivity = 0.90) [27]. The caregiver version of the PSRS also demonstrated excellent internal consistency (pain = 0.83; broad sensory hypo-reactivity = 0.90; broad sensory hyper-reactivity = 0.93) in a sample of individuals with autism spectrum disorders (ASD) [14]. The self-report version was used in the present study.
- Sensory Over-Responsivity Scales (SOR-Scales): The SORS assesses sensory hyper-reactivity to auditory, tactile, visual, olfactory, and taste stimuli. This tool was adapted from a measure used with a general community sample in a survey study [28]. It consists of rating scales addressing distress and impairment in relation to both auditory and tactile over-reactivity [29]. Each SORS subscale comprises four questions, with responses being provided along on a scale ranging from 0 to 4. Overall scores range from 0 to 80. Overall scores for each subscale are calculated separately and range from 0 to 16, with higher scores indicating greater severity. Cronbach alpha outcomes evaluating the internal consistency of the SORS overall and of its subscales indicated strong internal consistency when used in a sample from the United States (SOR-total = 0.93; SOR-hearing = 0.89; SOR-touch = 0.88; SOR-smell = 0.90; SOR-sight = 0.94; SOR-taste = 0.88) and in a sample from Spain (hearing = 0.89; touch = 0.86; smell = 0.91; sight = 0.90; taste = 0.86) [30].
- Obsessive–Compulsive Inventory–Revised (OCI-R): The OCI-R is an 18-item self-report questionnaire that assesses obsessive–compulsive symptom severity using a five-point Likert scale ranging from 0 (not at all) to 4 (very much). The OCI-R is comprised of six factors that represent the following symptom domains: checking, ordering, neutralizing, washing, obsessing, and hoarding [31]. Each factor is composed of three items, with possible scores ranging from 0 to 12. Overall, the measure has demonstrated good internal consistency when used in different countries (Cronbach’s α values ranging from 0.81 to 0.95 [32,33,34]).

### 2.5. Procedure

Participants completed all study measures via an online survey developed using LimeSurvey (LimeSurvey GmbH, Hamburg, Germany). At the beginning of each questionnaire, participants were required to input a unique code produced by LimeSurvey, as well as a valid email address for future study participation. All codes and emails were reviewed to guarantee that participants could not respond more than once. Full instructions were provided for the completion of all instruments. Approximately 20 min were required to complete all instruments. Tests were administered by experienced psychologists who provided instructions and individual assistance. Participants completed all procedures in their classrooms. A researcher remained in the classroom throughout questionnaire administration to assist students who experienced difficulties.

### 2.6. Data Analyses

Two subsamples, specifically, sample 1 (*n* = 624) and sample 2 (*n* = 623), were randomly selected from the overall set of observations (*N* = 1247). R, a free statistical software program, was used for all analytical processes (version 6.3). The performance of the instrument under study was examined according to skewness and kurtosis estimates and floor and ceiling effects. According to Ferrando, Lorenzo-Seva, Hernández-Dorado, and Muñiz [24] and Lloret-Segura, Ferreres-Traver, Hernández-Baeza, and Tomás-Marco [25], assumptions of normal distribution cannot be fulfilled when skewness and kurtosis coefficients are below −1.5 or above 1.5. Further, when more than 15% of participant responses correspond to extremely low or high response categories, floor and ceiling effects are deemed to be present [35,36]. Data were treated ordinally in accordance with criteria outlined by Rhemtulla et al. [37]. In subsample 1, EFA was performed to assess the instrument’s structure. The suitability of EFA was examined in accordance with Kaiser–Meyer–Olkin (KMO) (≥0.70 being acceptable [38]), the Bartlett test for sphericity (*p* < 0.05 being acceptable [39]), and coefficients of determination (values close to 0 being acceptable [24,25]) outcomes. Horn’s parallel analysis [24,25,40] was utilized to ascertain the number of factors. Estimates were made in line with the unweighted least squares (ULS) approach, which is advised for categorical variables when the normality assumption is broken, and Promax rotation was also used. EFA was performed using the “psych” package [41]. Criteria for item selection and refinement were based on saturation > 0.30 and exclusion of Heywood instances (saturation ≥ 1) [42]. The structure derived following EFA for the GSSS was then compared with the one-factor structure obtained by Martínez-González, Cervin, and Pérez-Sánchez [14] in children and adolescents using the weighted least square mean and variance-adjusted (WLSMV) method, which is advised for ordinal variables [43]. This analysis was conducted using the CFA of data gathered from subsample 2 using the “Lavaan” package [44]. Root mean square error of approximation (RMSEA), Tucker–Lewis (TLI), and comparative fit (CFI) indices were used to evaluate model fit. Model fit is deemed to be acceptable with CFI, TLI, and RMSEA values of >0.90, >0.90, and <0.06 [35,45], respectively. Three suggested statistical adjustments were made: (1) congeneric; (2) tau-equivalent; and (3) correlated error (modification indices > 35,000). In accordance with Brown [46]; Ferrando, Lorenzo-Seva, Hernández-Dorado, and Muñiz [24]; and Lloret-Segura, Ferreres-Traver, Hernández-Baeza, and Tomás-Marco [25], amongst others, in models containing Heywood cases, <35,000 correlated errors and negative variances were rejected. Internal consistency was evaluated for the overall sample through ordinal alpha coefficients, as such estimates yield more accurate outcomes when using categorical data. Acceptable dependability is indicated through α coefficients that are ≥0.70 [47,48]. The questionnaire was administered again four weeks after its first administration, and test–retest reliability (*n* = 45) was assessed according to the interclass correlation coefficient (ICC). In accordance with Martínez Pérez and Pérez Martin [49], an ICC value of ≥0.60 was deemed to be acceptable. Product–moment correlations between variables and items derived from the GSSS and those corresponding to the PSRS, SORS, and OCI-R were examined to assess scale validity for hypothesis testing. According to Prinsen, Mokkink, Bouter, Alonso, Patrick, de Vet, and Terwee [35], correlations between instruments assessing related but distinct constructs should be between 0.20–0.50 to support scale validity for hypothesis testing. In accordance with Wu and Estabrook [50], four types of invariances of the measure, configured with the structure indicated through EFA and confirmed via CFA, as a function of sex (*n* = 1235), were assessed. Specifically, measure invariance was determined according to (a) configural invariance; (b) metric invariance; (c) scalar invariance; and (d) strict invariance. Cases classified as “others” were excluded from analysis. When evaluating different degrees of measurement invariance, differences of ΔCFI ≤ 0.010 and ΔRMSEA ≤ 0.015 were deemed unimportant [51]. A more restrictive model may provide a better fit to the data than a less constrained model for indices that are penalized by a lack of parsimony [52]. A total of 20% of instrument items will be suppressed in the event that the next pre-specified threshold is not reached [51]. Finally, descriptive statistics and percentiles pertaining to the GSSS were calculated according to sex, excluding cases providing the response of “other”.

### 2.7. Ethical Considerations

All participants willingly agreed to participate in the present study. In the case of participants aged between 17 and 18 years, authorization for their participation in the study was obtained from their parents or legal guardians in accordance with the Declaration of Helsinki. The present study was approved by the Ethics Committee of the University of Alicante in Spain (reference number: UA-2019-10-04).

## 3. Results

### 3.1. Socio-Demographic and Clinical Characteristics of the Sample

Sociodemographic characteristics of the sample are presented in Table 1. A total of 1247 individuals with a mean age of 22.17 ± 7.19 years were evaluated, of which 72% were female. A total of 95.5% of the sample were of Spanish nationality (Valencian Community, Regions of Murcia, Aragon, and Andalusia). The presence of gastrointestinal problems in the sample is illustrated in the Supplementary Material (Table S1). It is worth noting that findings indicate that 23% of the present sample suffered from infectious diarrhea, 19.7% from stomach discomfort, 10.6% from dyspepsia, and 11.1% from gastroesophageal reflux.

### 3.2. Psychometric Assessment

Table 2 presents outcomes pertaining to the performances of instrument items. Floor effects, skewness, and kurtosis were observed, indicating that data were ordinal in nature.

#### 3.2.1. Exploratory Factor Analysis

**Figure 1:**
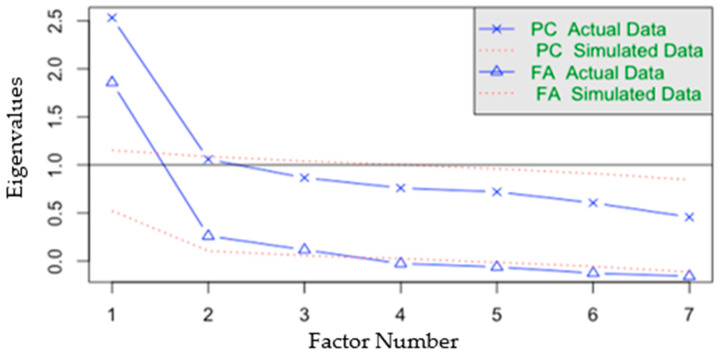
Factor extraction plot of the GSSS.

Horn’s parallel analysis was used for factor extraction, which yielded two factors (Figure 1). EFA was then performed with the first set of seven items. This produced a KMO ≥ 0.70, Bartlett-associated *p*-value < 0.05, and a coefficient of determination near zero. No items were deleted based on previously established criteria.

Table 3 displays item factor loadings. A modest proportion of explained variance (36.19% for factor 1 and 15.11% for factor 2) was found for the GSSS.

#### 3.2.2. Confirmatory Factor Analysis

Table 4 presents CFA outcomes following model adjustments made in line with predetermined criteria.

**Figure 2:**
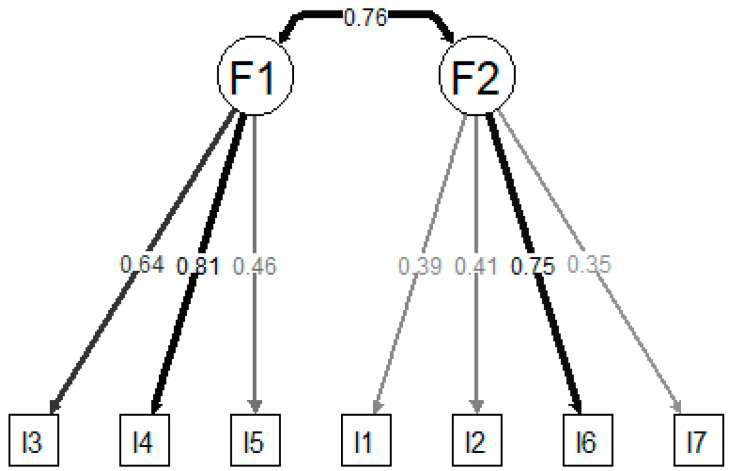
Factor loadings of the confirmatory factor analysis for the congeneric 2-factors model obtained in the EFA.

**Figure 3:**
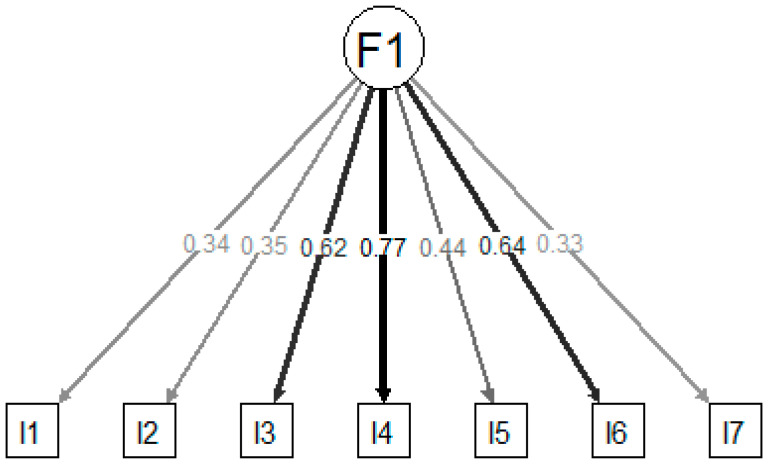
Factor loadings of the confirmatory factor analysis for the congeneric 1-factor model obtained in the EFA.

The congeneric two-factors model resulting from the EFA presented excellent fit, with factor loadings ranging between 0.35 and 0.81 (Figure 2). The congeneric single-factor model also presented excellent fit (Figure 3), although outcomes were slightly worse than those produced for the two-factors model. The tau-equivalent model presented only marginal fit and failed to achieve desired fit indices.

#### 3.2.3. Internal Consistency and Reliability

Internal consistency coefficients of 0.65 were produced for factor 1, 0.60 for factor 2, and 0.70 for the GSSS overall. Test–retest reliability of the GSSS at 4 weeks was 0.855 (95%CI [0.720–0.925]).

#### 3.2.4. Measurement Invariance

Findings regarding measurement invariance are presented in Table 5. Outcomes revealed that the metric measurement invariance of the scale as a function of sex can be assumed, as model fit was not reduced, in any instance, by a ΔCFI ≤ 0.010 or a ΔRMSEA ≤ 0.015. In order to examine whether partial scalar invariance was achieved, the item with the highest modification index and standardized parameter change (X^2^) was unrestrained within the model (item 5). Nonetheless, it was not possible to achieve scalar measurement invariance. Thus, Table 5 presents outcomes with the inclusion of all items.

#### 3.2.5. Hypothesis Testing for Construct Validity

Product–moment correlation outcomes pertaining to associations between factors corresponding to the GSSS and those pertaining to the PSRS, SOR, and OCI-R can be seen in Table 6. Overall, PSRS, SOR, and OCI-R scores were positively correlated with GSSS scores (r = 0.182 to 0.997; *p* < 0.01), with outcomes being in the expected direction and of the expected magnitude.

#### 3.2.6. GSSS Descriptive Statistics

Table S2 in Supplementary Materials presents means and percentiles pertaining to GSSS items for the overall sample and according to sex. Significant sex differences are observed.

## 4. Discussion

The main aim of the present study was to assess the psychometric properties of the GSSS in a Spanish sample of neurotypical adolescents and young adults. Study findings demonstrate that the GSSS is a tool with acceptable and promising psychometric properties.

Firstly, reports of GS made in the present study are highly similar to those made in previously conducted studies in a neurotypical adolescent population using the GSSS [22]. Likewise, findings regarding the prevalence of FGIDs coincide with those of previously conducted research [10,53]. Specifically, the most common symptoms are diarrhea, stomach discomfort, dyspepsia, and gastroesophageal reflux in adults with typical development. For example, an incidence of dyspepsia of around 10% was reported [53], alongside a 10% to 20% incidence of gastroesophageal reflux [10], in adults with typical development. Further, findings indicate sex differences in GS, with GSSS reports suggesting that females suffer from more severe GS compared to men. This finding is consistent with that reported by previous research [3,7,8].

Secondly, over the last few years, research on GS has largely focused on the pediatric population and on neurodevelopmental disorders. However, GS also emerges during adulthood and tends to coincide with a reduction in microbial diversity in the intestine (e.g., Faecalibacterium, Bacteroidaceae, and Lachnospiraceae) [54]. Furthermore, increasing age increases the likelihood of presenting with GS [55]. It is, therefore, necessary to examine GS during adulthood. In this sense, the GSSS is one of the first instruments that allows the analysis of GS at different evolutionary periods.

Thirdly, EFA outcomes suggest that the items of the GSSS pertain to two distinct dimensions. In this regard, present findings suggest that the first factor is related to the abdomen and includes items pertaining to abdominal pain, gas, and constipation (abdominal pain and defecation disorders), whilst the second factor is associated with the expulsion of ingested food (vomiting, defecation in inappropriate places, diarrhea, and rumination), in other words, functional nausea and vomiting disorders. These findings align with Rome IV criteria [1] and reports of a previously conducted study with a clinical sample with ASD [14]. In line with previous research, CFA revealed an excellent model fit when applying both a bidimensional and unidimensional structure, although the latter exhibited slightly poorer fit indices [14,22]. Additionally, ordinal alpha coefficient outcomes and test–retest reliability coefficients were acceptable and were similar to those found when using other instruments that are similar to the GSSS [18].

Fourthly, the measurement invariance of the GSSS was achieved as a function of sex. This finding is of great importance, as it is critical to ensuring that the instrument is equally reliable and valid for both males and females. This finding makes meaningful comparisons possible across sex, allowing researchers to reach reliable conclusions regarding sex differences [50]. In this context, evidence of measurement invariance decreases the risk of bias when administering the GSSS, as it ensures that any reported sex disparities reflect true differences in GS rather than measurement artefacts.

Fifthly, with regards to discriminant–convergent validity and utility of the instrument for hypothesis testing, a notable limitation of previous research examining GS scales pertains to the failure to perform convergent validity analysis. Indeed, existing studies tend to analyze discriminant relationships rather than convergent ones [18,56]. In the present work, a hypothesis was proposed that a relationship existed between GS and other variables, such as sensory reactivity. In this sense, the initially proposed hypothesis was confirmed. Significant correlations, with some being weak and others being strong, were observed between GSSS scores and the hypo-reactivity and hyper-reactivity dimensions comprised by the PSRS. Further, significant positive, albeit weak, correlations were identified between the GSSS, SORS, and OCI-R. These findings align with those reported in previous research and indicate the existence of a relationship between sensory reactivity, pain, and GS [12,13]. As in previous studies, the strongest correlation was found between tactile hypo-reactivity and the GSSS [22]. This finding seems to indicate a connection between cutaneous stimuli and the sensory circuits of the intestine [15].

Finally, the present research has a number of strengths and limitations that should be acknowledged. On the one hand, the GSSS exhibits acceptable psychometric properties and stands out as one of the first instruments focused on evaluating GS to achieve measurement invariance. This will be of great practical significance when it comes to interpreting group differences. On the other hand, it is important to note that the sample was highly homogeneous in terms of sex and age. In this regard and similarly to that reported by Crowell, Umar, Lacy, Jones, DiBaise, and Talley [18], a higher proportion of females characterized the present sample. This could have had an impact on outcomes when analyzing differences in GS as a function of sex. Future studies could analyze the psychometric properties of the GSSS in the population with FGIDs, as well as explore the psychometric properties of the GSSS from the Item Response Theory paradigm.

## 5. Conclusions

The GSSS provides a brief assessment tool to examine the severity of GS within adolescent and young adult populations. The psychometric properties of the GSSS, including factor structure, internal consistency, reliability, measure invariance, and validity for hypothesis testing were found to be acceptable to good. The GSSS provides a tool that may be useful for medical professionals when diagnosing FGIDs. It represents a new contribution to the evaluation of GS through self-reporting.

## Figures and Tables

**Table 1:** Sociodemographic characteristics of the total sample.

Variables	Total (*n* = 1247)
Age	
	22.17 (7.19) *
**Sex *n* (%)**	
Female	898 (72.0)
Male	337 (27.0)
Other	12 (1.2)
**Country/region of origin *n* (%)**	
Spain	1190 (95.0)
Rest of Europe	14 (1.2)
America	29 (2.5)
Africa	12 (1.1)
Asia	2 (0.2)

Note: * mean (standard deviation).

**Table 2:** Item performance of the GSSS.

Items	Min	Max	M (SD)	Skewness	Kurtosis	F.E (%)	C.E (%)
1. Regurgitation or rumination	0	3	0.18 (0.42)	2.39	5.64	1051 (83.9)	1 (0.1)
2. Vomiting	0	3	0.19 (0.46)	2.71	8.26	1051 (83.9)	5 (0.4)
3. Gas	0	3	0.65 (0.76)	1.04	0.66	625 (49.9)	32 (2.6)
4. Abdominal pain	0	3	0.54 (0.79)	1.40	1.28	766 (61.1)	40 (3.2)
5. Constipation	0	3	0.45 (0.74)	1.74	2.53	847 (67.6)	39 (3.1)
6. Diarrhea	0	3	0.30 (0.59)	2.24	5.30	956 (76.3)	17 (1.4)
7. Defecation in inappropriate places	0	3	0.07 (0.32)	5.03	27.54	1183 (94.4)	2 (0.2)

Notes: F.E = floor effect; C.E = ceiling effect; M = mean; SD = standard deviation; Min = minimum; Max = maximum.

**Table 3:** Results of exploratory factor analysis of the GSSS.

Items	Factor 1	Factor 2
1. Regurgitation or rumination		0.387
2. Vomiting		0.515
3. Gas	0.565	
4. Abdominal pain	0.801	
5. Constipation	0.415	
6. Diarrhea		0.619
7. Defecation in inappropriate places		0.517
**Explained variance %**	36.19	15.11
**Factor Correlations**		
Factor 1	1	
Factor 2	0.628	1

**Table 4:** Results of confirmatory factor analysis of the GSSS.

	Models	*χ*2	*df*	RMSEA (90% CI)	CFI	TLI
**2-Factors Model after EFA**	TM	102.394	18	0.085 (0.069–0.102)	0.824	0.795
	CM	28.052	13	0.007 (0.000–0.041)	0.999	0.999
**1-Factor Model**	TM	202.978	20	0.126 (0.111–0.142)	0.570	0.549
	CM	41.497	14	0.027 (0.000–0.051)	0.986	0.979

Notes: RMSEA = root mean square error of approximation; CFI = comparative fit index; TLI = Tucker–Lewis Index; CI = confidence interval; TM = tau-equivalent model; CM = congeneric model.

**Table 5:** Measurement invariance.

2-Factors Model	X^2^	gl	CFI	ΔCFI	RMSEA (90% CI)	ΔRMSEA
**Configurational**	52.283	26	0.942	-	0.040 (0.024–0.056)	-
**Metric**	**39.535**	**31**	**0.981**	-	**0.021** **(0.000–0.039)**	-
**Scalar**	62.468	36	0.942	−0.039	0.035 (0.019–0.049)	0.014
**Strict**	93.854	43	0.888	−0.054	0.044 (0.032–0.056)	0.009
**1-Factor Model**						
**Configurational**	72.806	28	0.902	-	0.037 (0.027–0.048)	-
**Metric**	**53.200**	**34**	**0.958**	-	**0.029** **(0.012–0.044)**	-
**Scalar**	86.725	40	0.898	−0.06	0.044 (0.031–0.056)	0.015
**Strict**	118.250	47	0.844	−0.054	0.053 (0.041–0.065)	0.009

Notes: RMSEA = root mean square error of approximation; CFI = comparative fit index; CI = confidence interval.

**Table 6:** Hypothesis testing for construct validity.

		Factor 1	Factor 2	Total GSSS
**PSRS**	Pain	0.30 **	0.22 **	0.22 **
	Total Hypo	0.26 **	0.27 **	0.30 **
	Hypo-Tactile	0.93 **	0.69 **	0.99 **
	Hypo-Olfactory	0.21 **	0.21 **	0.24 **
	Hypo-Visual	0.18 **	0.18 **	0.21 **
	Hypo-Taste	0.19 **	0.24 **	0.24 **
	Hypo-Auditory	0.24 **	0.22 **	0.22 **
	Total Hyper	0.30 **	0.26 **	0.33 **
	Hyper-Tactile	0.26 **	0.23 **	0.23 **
	Hyper-Olfactory	0.24 **	0.22 **	0.27 **
	Hyper-Visual	0.19 **	0.20 **	0.22 **
	Hyper-Taste	0.18 **	0.20 **	0.21 **
	Hyper-Auditory	0.25 **	0.18 **	0.26 **
**SOR**	Touch	0.19 **	0.19 **	0.22 **
	Smell	0.19 **	0.18 **	0.21 **
	Sight	0.17 **	0.19 **	0.21 **
	Taste	0.15 **	0.18 **	0.18 **
	Hearing	0.24 **	0.20 **	0.20 **
**OCI-R**	Hoarding	0.18 **	0.22 **	0.23 **
	Checking	0.17 **	0.19 **	0.20 **
	Ordering	0.18 **	0.12 **	0.18 **
	Neutralizing	0.18 **	0.15 **	0.19 **
	Washing	0.18 **	0.19 **	0.22 **
	Obsessing	0.23 **	0.19 **	0.25 **

Notes: GSSS = Gastrointestinal Symptom Severity Scale; OCI-R = Obsessive–Compulsive Inventory–Revised; SOR = Sensory Over-Responsivity Scale; Total Hypo = total sensory hypo-reactivity; Total Hyper = total sensory hyper-reactivity; ** = *p* < 0.01.

## Data Availability

All data and materials regarding this study are available from the corresponding author upon reasonable and formal request through the University of Alicante.

## Supplementary Materials

The following supporting information can be downloaded at: https://www.mdpi.com/article/10.3390/jcm13061662/s1, Table S1. Presence of gastrointestinal disorders in the total sample. Table S2. Percentiles of the GSSS.
