# Supplementary material for: Psychometric Properties of the Gastrointestinal Symptom Severity Scale in a Sample of Adolescents and Young Adults
Source: J Clin Med. 2024 Mar 14;13(6):1662. doi: 10.3390/jcm13061662 (PMC10971376; doi:10.3390/jcm13061662)
Supplement: Supplementary file 1 [file jcm-13-01662-s001.zip › jcm-2912846-supplementary.pdf]

Supplementary Table S1.

Presence of gastrointestinal disorders in the total sample.

**Gastrointestinal disorders**	***n* (%)**
Infectious diarrhea	287 (23.0)
Nonspecific abdominal pain	246 (19.7)
Dyspepsia	132 (10.6)
Gastroesophageal reflux	138 (11.1)
Significant Flatulence	67 (5.4)
Irritable Bowel Syndrome	46 (3.7)
Dyschezia	18 (1.4)
Inflammatory Bowel Disease	13 (1.0)
Celiac Disease	11 (0.9)
Ulcerative Colitis	9 (.7)
Peptic Ulcer Disease	5 (.4)
Crohn's disease	4 (.3)

Supplementary Table S2.

Percentiles of the GSSS.

	Percentile	Severity level of gastrointestinal symptoms	Factor 1	Factor 2	Total SSGS
**Total Sample**	29	Very Low	0	0	1
	49	Low	1	0	2
	69	Medium-low	2	1	3
	79	Medium-High	3	1	4
	89	High	4	2	5
	99	Very High	7	6	12
**M (SD) Total Sample**			1.63 (1.74)	.73 (1.21)	2.37 (2.54)
**Females**	29	Very Low	0	0	1
	49	Low	1	0	2
	69	Medium-Low	2	1	3
	79	Medium-High	3	1	4
	89	High	4	2	6
	99	Very High	8	6	12
**M (SD) Females**			1.79 (1.82)	.76 (1.24)	2.55 (2.64)
**Males**	29	Very Low	0	0	0
	49	Low	1	0	1
	69	Medium-Low	2	1	3
	79	Medium-High	2	1	3
	89	High	3	2	4
	99	Very High	6	6	10
**M (SD) Males**			1.18 (1.38)	.66 (1.09)	1.84 (2.13)

**Contrast Statistics between sex (Effect Size)**	**P value**	**Hedges' g**
Total SGGS Females vs. Total SGGS Males	$p < .05$	.28
Factor 1 SSGS Females vs. Factor 1 SGGS Males	$p < .05$	.35
Factor 2 SSGS Females vs. Factor 2 SGGS Males	$p > .05$	.08

Notes. M=Mean; SD= Standard Deviation.
